# A systematic review of the effectiveness of employer‐led interventions for drug misuse

**DOI:** 10.1002/1348-9585.12133

**Published:** 2020-06-13

**Authors:** Maxwell O. Akanbi, Cassandra B. Iroz, Linda C. O'Dwyer, Adovich S. Rivera, Megan Colleen McHugh

**Affiliations:** ^1^ Institute for Public Health and Medicine Feinberg School of Medicine Northwestern University Chicago USA; ^2^ Galter Health Sciences Library and Learning Center Feinberg School of Medicine Northwestern University Chicago IL USA; ^3^ Department of Emergency Medicine Feinberg School of Medicine Northwestern University Chicago IL USA

**Keywords:** illicit drugs, intervention, opioids misuse, systematic review, workplace

## Abstract

**Aims:**

Employers in the United States incur substantial costs associated with substance use disorders. Our goal was to examine the effectiveness of employer‐led interventions to reduce the adverse effects of drug misuse in the workplace.

**Methods:**

We conducted a systematic review of studies that evaluated the effectiveness of recommended workplace interventions for opioids and related drugs: employee education, drug testing, employee assistance programs, supervisor training, written workplace drug‐free policy, and restructuring employee health benefit plans. We searched PubMed MEDLINE, EMBASE (embase.com), PsycINFO (Ebsco), ABI Inform Global, Business Source Premier, EconLit, CENTRAL, Web of Science (Thomson Reuters), Scopus (Elsevier), Proquest Dissertations, and Epistemonikos from inception through May 8, 2019, with no date or language restrictions. We included randomized controlled trials, quasi‐experimental studies, and cross‐sectional studies with no language or date restrictions. The Downs and Black questionnaire was used to assess the quality of included studies. The results were reported using the Preferred Reporting Items for Systematic Reviews and Meta‐Analysis (PRISMA) guidelines.

**Results:**

In all, 27 studies met our inclusion criteria and were included in the systematic review. Results were mixed, with each intervention shown to be effective in at least one study, but none showing effectiveness in over 50% of studies. Studies examining the impact of interventions on workplace injuries or accidents were more commonly reported to be effective. Although four studies were randomized controlled trials, the quality of all included studies was “fair” or “poor.”

**Conclusions:**

Despite the opioid epidemic, high‐quality studies evaluating the effectiveness of employer‐led interventions to prevent or reduce the adverse effects of substance use are lacking. Higher quality and mixed methods studies are needed to determine whether any of the interventions are generalizable and whether contextual adaptations are needed. In the meantime, there is a reason to believe that commonly recommended, employer‐led interventions may be effective in some environments.

## 1 INTRODUCTION

The United States (US) is facing its worst opioid crisis in history.^1^, ^2^ Despite efforts to mitigate the epidemic, drug overdoses were responsible for approximately 70 237 deaths in 2017 (47 600; 67.8% from opioids), representing a 9.6% increase from 2016.^1^, ^2^, ^3^ Substance use disorder, which includes the misuse of opioids, has a significant impact on the workforce. A recent analysis of the 2012‐2014 National Survey on Drug Use and Health indicated that 20.2 million adults had a self‐reported substance use disorder, and more than 60% were employed.^4^ Given the large number of employees reporting a substance use disorder, employers are incurring a significant portion of the estimated $400 billion annual cost of substance abuse,^4^ including costs associated with absenteeism, occupational injuries,^5^ turnover, and health care.^4^ The need for effective interventions to reduce the burden of substance use, including misuse of opioids, in the workplace is urgent and could potentially target a large proportion of users.

The Substance Abuse and Mental Health Services Administration (SAMHSA) of the US Department of Health and Human Services recommends five types of employer‐initiated interventions.^6^ These interventions include the following: establishment of a clear written workplace policy on substance use; employee education to improve knowledge about opioids and other potentially addictive medication; training of supervisors to keep them updated with the most recent workplace drug policies and identification of signs of impairment among other things; employee assistance programs to support confidential treatment of affected workers adoption of drug‐testing policies; and redesigning health benefits to improve access to health services. In some instances, interventions are extended to immediate family members of employees because of the known negative impact of ill health among employees’ family members on workplace productivity.

Despite the increase in the number of organizations adopting interventions to deter employees from the misuse of prescription medication and illegal drugs,^7^, ^8^ critical evaluation of the effectiveness of these interventions is sparse. Reviews are either dated ^9^, ^10^, ^11^ or focused on a particular occupational group,^12^ drug,^12^ intervention,^12^, ^13^, ^14^ or outcome.^12^, ^14^ Prior reviews have concluded that there is weak evidence to support the effectiveness of recommended interventions to deter employees from illicit drug use. However, the opioid epidemic has generated renewed interest in this field as employers seek the best ways to insulate the workplace from the adverse effects of drugs. Given the limitations of previous reviews, our goal was to systematically review the evidence of the effectiveness of recommended employer‐initiated interventions aimed at reducing the negative impact of major drugs of abuse in the workplace.

## 2 MATERIALS AND METHODS

We used the Preferred Reporting Items for Systematic Reviews and Meta‐Analysis (PRISMA)^15^ guideline for reporting this systematic review and registered the review protocol in the International prospective register of systematic reviews, PROSPERO (Registration number: CRD42019132681).

### 2.1 Search strategy

We searched PubMed MEDLINE, EMBASE (embase.com), PsycINFO (Ebsco), ABI Inform Global, Business Source Premier, EconLit, CENTRAL, Web of Science (Thomson Reuters), Scopus (Elsevier), Proquest Dissertations, and Epistemonikos from inception through May 8, 2019, with no date or language restrictions. Terms used in the search included *workplace, employer, employee, substance‐related disorders, substance abuse, substance misuse, and interventions*. A full list of the search strategies is outlined in Appendix A.

### 2.2 Inclusion and exclusion criteria

We included randomized controlled trials (RCTs), quasi‐experimental studies, cohort studies, cross‐sectional studies, and pre‐post studies that investigated the effectiveness of an employer‐initiated intervention to reduce the adverse effects of opioids and other drugs of addiction**.** We focused on the six categories of employer‐initiated interventions recommended by SAMHSA and other related organizations^6^, ^16^, ^17^: employee education, drug testing (random, post‐accident and reasonable suspicion), employee assistance programs (EAP), supervisor training, written workplace drug‐free policy, and restructuring of employee health benefit plans.^6^ We excluded studies that exclusively investigated pre‐employment drug screening, as our focus was on interventions targeted to employees. We included articles focused on the eight groups of drugs identified during the 2015‐2017 National Surveys on Drug Use and Health as the major drugs of abuse in the United States^18^ (Appendix B). We included articles that reported outcomes related to drug use or their direct effects, including accidents and injuries, absenteeism, healthcare utilization, cost, and other measures of productivity. Interventions were considered to be effective if they reduced drug use or the adverse effects of drug use. We excluded case reports, case series, editorials, commentaries, and publications that investigated workplace interventions only for alcohol abuse or tobacco use.

### 2.3 Data collection and processing

Search results were saved into EndNote files by the librarian (LCO) and transferred into Covidence^19^ for subsequent processing. Two reviewers (MOA and CBI) independently performed the title and abstract screening, and the full‐text screening. Conflicts were resolved through consensus. Extraction of data from included studies was carried out independently by three reviewers (MOA, ASR, and CBI; two reviewers per article) using a data extraction template designed by the investigators and embedded into Covidence. Information extracted included: year of publication, the country where the intervention took place, study design, study sample, number of participants, intervention type, outcome measures, and effectiveness of the intervention. For study outcomes, we selected results from fully adjusted models, when available. For studies that reported outcomes for several illicit drugs, we selected outcomes of opioids. We selected the most rigorous assessment of the reported outcomes.

### 2.4 Methodical quality assessment

We assessed the methodical rigor of the included studies using the modified Downs and Black checklist for randomized and non‐randomized studies for healthcare.^20^, ^21^, ^22^, ^23^ The checklist has 27 items, with a total possible score of 28. Papers were rated excellent if they scored above 25, good if they scored between 20 and 25, fair if they scored between 15 and 19, and poor if they scored <15.^24^ Each study was assessed by two independent investigators, and discrepancies in scoring were resolved through consensus.

## 3 RESULTS

### 3.1 Study selection

**FIGURE 1:**
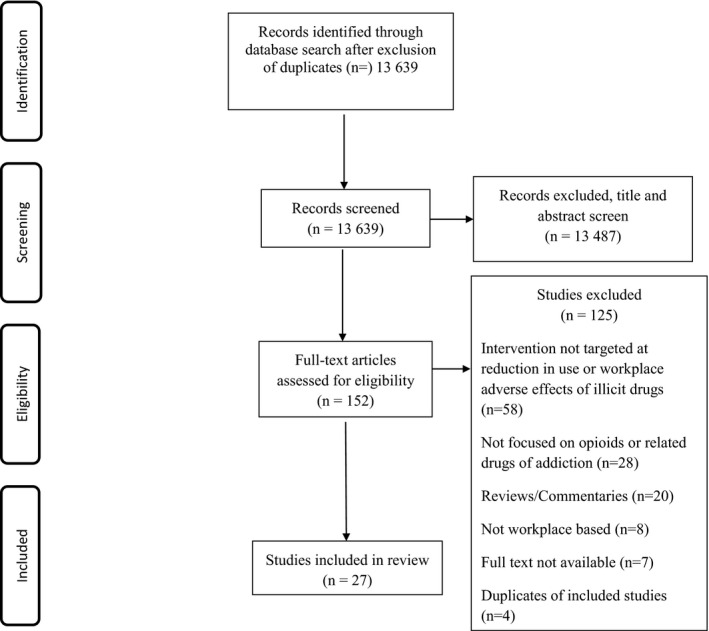
PRISMA Flow chart for literature search

We identified 21 620 titles (PubMed MEDLINE 3014; EMBASE [embase.com] 4430; PsycINFO [Ebsco] 962; ABI Inform Global 1793; Business Source Premier 120; EconLit 45; CENTRAL 3273; Web of Science [Thomson Reuters] 1603; Scopus [Elsevier] 5551; Proquest Dissertations 327; and Epistemonikos 502). After the removal of duplicates, 13 639 title and abstracts were screened. Based on the review of titles and abstracts, 13 487 papers unrelated to the topic of interest were excluded. The full‐text review was conducted on 152 articles out of which 27 were ultimately included in the review.^25^, ^26^, ^27^, ^28^, ^29^, ^30^, ^31^, ^32^, ^33^, ^34^, ^35^, ^36^, ^37^, ^38^, ^39^, ^40^, ^41^, ^42^, ^43^, ^44^, ^45^, ^46^, ^47^, ^48^, ^49^, ^50^, ^51^ The list of excluded studies and reasons for exclusion are shown in Appendix C. The level of concordance of the reviewers during the initial full‐text review was 83%. Figure 1 shows the study flowchart.

### 3.2 Characteristics of studies

Four^25^, ^28^, ^29^, ^43^ of the 27 included studies were RCTs. Nine studies were quasi‐experimental studies, of which eight were interrupted time‐series analyses,^32^, ^34^, ^37^, ^39^, ^40^, ^42^, ^47^, ^49^ and one was historically controlled.^27^ In all, 14 studies were observational studies, of which seven were cross‐sectional,^26^, ^31^, ^33^, ^41^, ^44^, ^46^, ^50^ and seven were cohort studies.^30^, ^35^, ^36^, ^38^, ^45^, ^48^, ^51^ The majority of the studies (23/27; 85%) were carried out among employees in the United States. Australia, Canada, Portugal, and Spain had one study each. The most common independent intervention was drug testing, which had 12 independent analyses from 11 studies.^26^, ^30^, ^31^, ^33^, ^35^, ^37^, ^38^, ^39^, ^42^, ^45^, ^50^ Seven analyses from five studies evaluated the effectiveness of EAPs,^26^, ^27^, ^39^, ^49^, ^51^ while six studies investigated the impact of employee education.^25^, ^26^, ^28^, ^29^, ^39^, ^43^ Less commonly evaluated single interventions were written workplace drug‐free policies with five effectiveness evaluations^26^, ^33^, ^39^, ^44^, ^50^ and restructuring of employee benefits, with three evaluations from two studies.^34^, ^48^ Four studies evaluated multiple interventions independently,^26^, ^33^, ^39^, ^48^ and six studies evaluated multiple interventions collectively.^32^, ^36^, ^40^, ^44^, ^47^, ^51^ The most frequently assessed outcomes were the reduction in illicit drug use and reduction in workplace accidents. Other reported outcomes included direct costs (eg, cost of injuries, cost of mental health services, company claims), absenteeism, involuntary turnover, and healthcare utilization (Table 1).

**TABLE 1:** Characteristics of studies evaluating workplace interventions for opioid use disorder and related conditions

Study	Study design	Intervention(s)	Country	Industry	Number of participants	Number of companies/sites	Outcomes evaluated (measurement method)	Quality assessment
*Employee education*								
Brochu 1988^25^	Randomized controlled trial	Employee education	Canada	Not reported	435	1 site	Illicit drug use (self‐report using randomized response technique)	Fair
Cook et al 2000^28^	Randomized controlled trial	Employee education	USA	Insurance	424	1 site	Drug use (self‐report)	Poor
Cook 2004^29^	Randomized controlled trial	Employee education	USA	Construction	201	5 sites	Drug and marijuana use (self‐report and urine and hair tests)	Fair
Patterson 2005^43^	Randomized controlled trial	Employee education	USA	Construction (37% of participants), small aircraft pilots and maintenance (4%), bus drivers (19%), materials moving (10%), hotels (6%), restaurants (including bars and cafeterias; 16%), and other services (home health care, car washes, concessions; 9%).	539	Survey of small business employees	Use of over‐the counter drugs for unwinding (self‐report)	Fair
*Drug testing*								
French 2004^31^	Cross‐sectional	Suspicion‐based and random drug testing	USA	National Survey	15 400	National survey	Drug use (self‐report)	Fair
Marques 2014^37^	Retrospective cohort study	Random drug testing	Portugal	Transportation (railway)	3801	1 company	Workplace accidents (routinely collected data)	Fair
Messer 1996 ^38^	Retrospective cohort study	Random drug testing	USA	Transportation	16 739	1 agency	Rates of vehicular accidents and passenger injuries (routinely collected data), Substance use (biochemical tests)	Fair
Lockwood 2000^35^	Interrupted time series with no control	Random drug test	USA	Hotel	Not reported	1 hotel	Workplace accidents (routinely collected data)	Poor
Ozminkowski 2003^42^	Interrupted time series with no control	Random drug testing	USA	Manufacturing	1791	15 sites	Total medical expenditures, Expenditure for substance abuse or related treatment, Workplace injuries (routinely collected data)	Fair
Shepard 1998^46^	Cross‐sectional	Random drug testing	USA	Computer and communications equipment	Not reported	63 companies	Productivity per worker defined by sales (routinely collected data)	Poor
Schofield 2013^45^	Retrospective cohort study	Random drug testing	USA	Construction	185 808 952 h of employee time at risk, representing approximately 92 882 full‐time equivalent employees (FTE)	1360 companies	Injury rates, Injury severity, Medical claims (routinely collected data)	Fair
Morantz 2008^41^	Controlled interrupted time series	Post‐accident drug testing	USA	Retail	Not reported		Workers/compensation claims, First aid reports (routinely collected data)	Fair
Feinauer 1993^30^	Retrospective cohort study	Post‐accident and reasonable cause drug testing	USA	All (with a subcategory for manufacturing)	Not reported	48 facilities	Change in OSHA injury rate (routinely collected data)	Fair
*Employee assistance program*								
Castro 2000 ^27^	Historically controlled trial	EAP	USA	Electrical and gas installation	52	1 company	Accidents, Sick leave hours Workers’ compensation claims (routinely collected data)	Poor
Sweeney 1995^49^	Controlled interrupted time series	EAP	USA	Manufacturing	954	1 site	Mental health/chemical dependency claims/person/month, Cost of mental health/chemical dependency claims/person/month (routinely collected data)	Fair
Waehrer 2016^50^	Cross‐sectional	EAP	USA	Various non‐agricultural	1405	National survey	Non‐fatal workplace injuries (survey)	Fair
*Restructuring employee health benefit plans*								
LoSasso 2004^34^	Retrospective cohort study	Restructuring of Employee Health Benefit Plans	USA	Not specified	656	399 employers	Mental health and substance abuse treatment utilization (routinely collected data)	Fair
Sturm 2000^48^	Retrospective cohort study	Restructuring of Employee Health Benefit Plans	USA	Not specified	408 663 person‐years (1 142 273 member‐years including dependents)	49 employers	Substance abuse treatment utilization and cost: inpatient and outpatient (routinely collected data)	Fair
*Multiple interventions assessed separately*								
Carpenter 2007^26^	Cross‐sectional study	Employee education, Random drug testing, Written workplace policy, EAP	USA	For‐profit firms across the USA	57 397	National survey	Marijuana use (self‐report/national survey)	Fair
Miller 2015^39^	Cross‐sectional	Employee education, Drug testing, Written workplace policy, EAP	USA	National survey	24 230	National survey	Drug use including any prescription drug, pain relievers, stimulants and sedatives (self‐report)	Poor
Lee 2011^33^	Cross‐sectional	Drug testing, Written workplace policy	USA	All	2249	National survey	Misuse of prescription pain relievers (self‐report)	Poor
Sturm 2000^48^	Retrospective cohort study	Restructuring of Employee Health Benefit Plans	USA	Not specified	408 663 person‐years (1 142 273 member‐years including dependents)	49 employers	Substance abuse treatment utilization and cost: inpatient and outpatient (routinely collected data)	Fair
*Combined Interventions*								
Lockwood 1998^36^	Time‐series quasi‐experimental	EE + Drug testing + EAP + Supervisor training + written workplace drug‐free policy, EE + Drug testing + Supervisor training + written workplace drug‐free policy	USA	Hotel	>2340	5 hotels	Absenteeism, Injuries, Health insurance claims, Productivity, (routinely collected data)	Fair
Gómez‐Recasens 2018^32^	Non‐randomized single arm study	Employee education + random/suspected use/post‐accident drug testing	Spain	Construction	1103	12 work centers	Risky drug use (saliva drug test)	Fair
Miller 2007^40^	Controlled interrupted time series	Employee Education + EAP + Random drug testing)	USA	Transportation	Not reported		Injury rates, Cost of injuries	Fair
Spicer 2005^47^	Controlled interrupted time series	Employee education + Random drug testing	USA	Transportation	Not reported	5 companies	Injury rate (routinely collected reports)	Poor
Wickizer 2004^51^	Retrospective cohort study	Written workplace policy + Drug testing + EAP + Employee education	USA	Agriculture, Forestry, and Fishing, Mining, Construction, Manufacturing, Transportation and Public Utilities, Wholesale and Retail Trade, Finance, Insurance, and Real Estate, Services	Not reported	261 intervention companies and 20 215 control companies	Injury rate (routinely collected data)	Fair
Pidd 2016^44^	Cross‐sectional	Written workplace policy + Drug testing, Assistance with drug use + Employee education, Written workplace policy + Drug testing + Assistance with drug use	Australia	National population‐based survey	13 590	National survey	Illicit drug use (self‐report)	Poor

Abbreviations: EAP, employee assistance program, OSHA, Occupational Safety and Health Administration, USA, United States of America.

### 3.3 Quality of studies

All of the included studies were rated either fair or poor, with scores ranging from 8/28 to 19/28 (Table 2). None of the studies met the threshold for “excellent” or “good” quality, based on the modified Downs and Black criteria.^20^ The majority of the studies (18; 66.7%) had total scores within the range for “fair quality,” while the remaining nine fell within the “poor quality” range. Of the four RCTs, two had scores within the “poor quality” range,^25^, ^28^ and the remaining two had scores within the “fair quality” range.^29^, ^43^ In general, the weakness in quality scores reflects poor scores for internal validity (high risk of bias or unmeasured confounders) and power estimation.

**TABLE 2:** Risk of Bias assessment of included studies based on the Downs and Black tool

Study ID	Score																												
	Reporting										External validity			Internal validity‐bias							Internal validity‐Confounding				Power			Total	Quality
Question number	1	2	3	4	5	6	7	8	9	10	11	12	13	14	15	16	17	18	19	20	21	22	23	24	25	26	27		
Brochu 1988^25^	1	1	1	1	2	0	1	0	0	0	1	0	1	0	1	1	1	1	0	0	1	1	1	0	0	0	0	16	Fair
Carpenter 2007^26^	1	1	1	1	2	1	1	0	0	0	1	0	1	0	0	1	0	1	0	0	0	1	0	0	1	0	0	14	Poor
Castro 2000^27^	1	1	1	0	0	1	1	0	1	1	0	0	0	0	0	1	0	0	1	1	1	0	0	0	0	0	0	11	Poor
Cook 2000^28^	1	1	1	1	2	0	0	0	0	0	0	0	0	0	0	1	1	1	1	0	1	1	1	0	0	0	0	13	Poor
Cook 2004^29^	1	1	1	1	2	1	1	0	1	0	0	0	1	0	0	1	1	1	1	1	1	1	0	0	0	0	0	17	Fair
Feinauer 1993^30^	1	1	1	1	0	0	0	0	1	0	1	0	1	0	0	1	1	1	1	1	1	1	0	0	1	1	0	16	Fair
French 2004^31^	1	1	1	1	2	1	1	0	0	0	1	1	1	0	0	1	0	1	0	0	0	1	0	0	1	0	0	15	Fair
Gómez‐Recasens 2018^32^	1	1	1	1	2	1	1	0	1	1	1	0	1	0	0	1	0	0	1	1	1	0	0	0	0	0	0	16	Fair
Lee 2011^33^	1	1	1	1	2	0	0	0	0	1	1	1	1	0	0	1	0	1	0	1	0	0	0	0	1	0	0	14	Poor
Lockwood 1998^36^	1	1	1	1	2	0	1	0	1	1	1	1	1	0	0	1	1	1	1	1	0	0	0	0	0	1	0	18	Fair
Lockwood 2000^35^	1	1	0	1	0	0	0	0	0	0	0	0	0	0	0	1	1	1	0	1	0	1	0	0	0	0	0	8	Poor
LoSasso 2004^34^	1	1	1	1	2	0	0	0	0	0	1	1	1	0	0	1	0	1	1	1	1	1	0	0	1	0	0	16	Fair
Marques 2014^37^	1	1	0	1	2	1	1	0	0	1	1	1	1	0	0	0	1	1	1	1	1	1	1	0	1	0	0	19	Fair
Messer 1996^38^	1	1	1	1	2	1	0	0	0	0	1	1	0	0	0	1	1	1	1	1	0	1	0	0	0	0	0	15	Fair
Miller 2007^40^	1	1	0	1	2	1	1	0	0	0	1	1	1	0	0	1	0	1	1	1	0	1	0	0	1	0	0	16	Fair
Miller 2015^39^	1	1	1	1	2	0	1	0	0	1	1	1	1	0	0	1	0	1	0	0	0	0	0	0	1	0	0	14	Poor
Morantz 2008^41^	1	1	1	1	2	1	1	0	0	0	1	0	1	0	0	1	1	1	1	1	0	1	0	0	1	0	0	17	Fair
Ozminkowski 2003^42^	1	1	1	1	2	0	1	0	0	1	0	1	1	0	0	1	1	1	0	1	0	0	0	0	1	0	0	15	Fair
Patterson 2005^43^	1	1	1	1	2	1	0	0	1	1	1	1	0	0	0	1	1	1	1	0	1	0	1	0	1	0	0	18	Fair
Pidd 2016^44^	1	1	1	1	2	0	0	0	0	1	1	1	1	0	0	1	0	1	0	0	0	0	0	0	1	0	0	13	Poor
Schofield 2013^45^	1	1	1	1	2	1	1	0	0	0	1	1	1	0	0	1	1	1	1	1	1	1	0	0	1	0	0	19	Fair
Shepard 1998^46^	1	1	0	1	2	0	0	0	0	0	0	0	0	0	0	1	0	1	1	1	0	0	0	0	0	0	0	9	Poor
Spicer 2005^47^	1	1	1	1	2	0	0	0	1	0	0	0	0	0	0	1	0	1	0	1	1	1	0	0	1	1	0	14	Poor
Sturm 2000^48^	1	1	1	1	2	0	0	0	0	0	0	0	1	0	0	1	1	1	1	1	1	1	0	0	1	0	0	15	Fair
Sweeney 1995^49^	1	1	1	1	2	1	0	0	1	1	0	0	1	0	0	1	1	1	1	1	1	1	0	0	0	1	0	18	Fair
Waehrer 2016^50^	1	1	1	0	2	1	1	0	0	0	1	1	1	0	0	1	0	1	0	1	1	0	0	0	1	0	0	15	Fair
Wickizer 2004^51^	1	1	1	1	0	1	1	0	1	0	1	1	1	0	0	1	1	1	1	1	1	1	0	0	0	1	0	18	Fair

### 3.4 Effectiveness of Interventions

Because some studies evaluated multiple interventions or outcomes, we identified 49 independent analyses of the effectiveness of recommended workplace interventions. A summary of the effectiveness of the interventions is provided in Table 3.

**TABLE 3:** Effectiveness of Workplace interventions for misuse of opioids and related drugs

Outcomes	Studies	Study design	Results	Comments	Quality
**A. Intervention: employee education**					
Illicit drug use	Brochu 1988^25^	Randomized‐controlled trial	Self‐reported marijuana or hashish use in the last 12 mo: Intervention 32%, Control 23% (variance = 0.05 and 0.02, respectively), *t* = 0.24; *P* > .01	Education did not result in the reduction of illicit drug use.	Fair
	Carpenter 2007^26^	Cross‐sectional	Self‐reported marijuana use in the last 30 d: aOR 0.791, SE 0.048, *P* < .01	21% lower odds of marijuana use.	Poor
	Cook 2000^28^	Randomized‐controlled trials	Self‐reported illicit drug use: Pre‐Intervention:16 using illicit drugs Post‐test 1:5/16, McNemar test *P* = .02 Post‐test 2:2/9, McNemar test *P* = NS	Data only presented for intervention group. Stress management education led to significant reduction in the use of illicit drugs in the short term (1 mo), but not long term (10 mo)	Poor
	Cook 2004^29^	Randomized‐controlled trial	Self‐reported illicit drug use in the past 30 d: Intervention 6%, Control 14% (*χ* ^2^ = 2.32, *P* = .128)	Education did not result in the reduction of illicit drug use	Fair
	Miller 2015^39^	Cross‐sectional	Self‐reported non‐medical prescription drug use in the last 30 d: aOR 0.98; 95% CI 0.85‐1.14, *P* = .834	No association between education and drug misuse	Fair
	Patterson 2005^43^	Randomized‐controlled trial	Likelihood to use over the counter drug to relax (Likert scale: 1‐5): Mean comparison, pre‐, and post‐intervention: Intervention 1:Pre 2.20, post 2.29; Intervention 2: Pre 2.30, post 2.15; Control: Pre 2.37, Post 2.26 ANOVA, *F* = 1.92, *P* > .05	Education did not result in the reduction of illicit drug use	Fair
**B. Intervention: drug testing**					
Illicit drug use	Carpenter 2007^26^	Cross‐sectional	Self‐reported marijuana use in the last 30 d: (AOR 0.697, SE 0.050) *P* < .01)	31% lower odds of marijuana use	Poor
	French 2004^31^	Cross‐sectional	Any drug use: 1. Any drug testing: *β* = −0.31, SE 0.06, *P* < .01 2. Suspicion‐based: *β* = −0.35 SE 0.08, *P* < .01 3. Random: β = −0.38, SE 0.10 *P* < .01	Lower rate of illicit drug use among employees at worksites with any drug testing, random drug testing or suspicion‐based drug‐testing program	Fair
	Lee 2011^33^	Cross‐sectional	Misuse of prescription pain relievers. Any drug testing: *β* = 0.2, SE 0.22 *P* = NS	No association between drug testing and misuse of prescription pain relievers	Poor
	Messer 1996^38^	Retrospective cohort study	Positive results on drug test: Non‐random drug test: Year 1 2.6%, Year 2:1.6%, Year 3 1.4%; 1.2% decline in year 3 compared to year 1. Random drug test: Year 1 2.3%, Year 2:2.1%, Year 3:1.5%, 0.8% decline in Year 3 compared to Year1	Introduction of random drug testing did not lead to a significant decline in positive drug tests compared to non‐random tests	Fair
	Miller 2015^39^	Cross‐sectional	Non‐medical prescription drug use in the last 30 d: aOR, 0.92, 95% CI 0.78‐1.07, *P* = .276	No association between drug testing and drug misuse	Fair
Work‐related Injuries	Feinauer 1993^30^	Retrospective cohort study	OSHA reportable accidents over 5 y: Any Drug testing: *β* = −1.220, SE −0.068, *t*: −0.509, *df*: 43, *P*: NS) Post‐accident drug testing: *β* = −2.823, SE −0.225, *t*: −2.792, *P* < .01) Reasonable cause drug testing: *β* = −0.163, SE: −0.014, *t*: −0.115, *P* > .05	Post‐accident drug testing was effective in reducing workplace accidents Any drug test or reasonable cause drug testing did not reduce accident rates	Fair
	Lockwood 2000^35^	Interrupted time series (no control)	OSHA reportable accidents: Pre‐employment drug test vs. pre‐employment + Random drug test. Pre‐intervention slope = 0.21 Post‐intervention slope = −0.04 Change in slope = *t* test = −2.70, *P* < .01	Introduction of random drug testing led to a reduction in OSHA reportable accidents	Poor
	Marques 2014^37^	Retrospective cohort study	Workplace accidents: Untested employees: 47.0% Random drug test: 19.4% Adjusted *P* < .001	Employees randomly selected for drug testing were less likely to have workplace accidents following the test, compared to untested employees	Fair
	Messer 1996^38^	Retrospective cohort study	Mean accidents rates/1 000 000 miles: Random drug test: 1.5%, Non‐random drug test: 1.9%, *P* = NS Passenger injury rates/100 000 miles: Random drug test: 3.9%, non‐random drug test: 5.2%, *t* (62) = 1.85, *P* = .045	A change from non‐random to random drug test led to a decline in passenger injuries, but not overall accidents	Fair
	Ozminkowski 2003^42^	Interrupted time series (No control)	Regression odds of a workplace accident: aOR: −0.5856; *P* = .0532	Random drug testing led to lower accident rates, but the change was not statistically significant	Fair
	Schofield 2013^45^	Retrospective cohort study	All workplace injuries: No program versus pre‐employment/post‐accident: RR = 0.85, CI = 0.72‐1.0, *P* = NS No program versus pre‐employment/post‐accident/random/suspicion: RR = 0.97 95% CI = 0.86‐1.10), *P* = NS	Drug testing was not associated with a significant reduction in workplace injuries	Fair
	Waehrer 2016^50^	Cross‐sectional	No work lost injuries: IRR 0.859, SE 0.062, *P* < .01 Injuries resulting in job loss: IRR 0.92, SE 0.054, *P* = NS	Drug testing was associated with a reduction in injuries that did not result in loss of work, but not injuries that resulted in work loss	Fair
Healthcare Cost	Morantz 2008^41^	Controlled interrupted time series	Total worker compensation claims: aOR = −0.123, SE 029, *P* < .01	Introduction of drug testing led to a significant decline in total worker compensation claims	Fair
	Ozminkowski 2003^42^	Interrupted time series (No control)	Any substance abuse or related expenditure: aOR = −1.0356, *P* = .3504	Random drug testing did not lead to a reduction in substance abuse or related expenditure	Fair
Productivity	Shepard 1998^46^	Cross‐sectional	Productivity: Log sales/employee Any drug testing:regression coefficient: −0.192, SE 0.077, *P* < .01 Pre‐employment drug test: regression coefficient: −0.16, SE 0.082, *P* < .05 Random drug test: regression coefficient: −0.285, SE 127, *P* < .02	Any form of drug testing was associated with a 19% reduction in productivity. Pre‐employment and random drug testing was associated with a 16% and 29% reduction in productivity, respectively	Poor
**C. Employee Assistant Programs**					
Illicit drug use	Carpenter 2007^26^	Cross‐sectional	Self‐reported marijuana use in the last 30 d: aOR 1.01, SE 0.064, *P* > .05	No association between EAP and illicit drug use	Poor
	Miller 2015 ^39^	Cross‐sectional	Self‐reported non‐medical prescription drug use: aOR 0.85, 95% CI 0.72‐1.00, *P* = .047	EAP was associated with 15% lower non‐medical prescription drug use	Fair
Work‐related Accident	Castro 2000^27^	Historically controlled trial	Number of Accidents: Mean number of accidents‐ Pre‐EAP: 2.22, SD 1.9 Post‐EAP: 1.0 (SD 1.32) Mean difference; −1.21 (SD 2.49), *t*‐value = −2.79; *P* = .009	Introduction of EAP led to a significant reduction in the number of workplace accidents	Poor
	Waehrer 2016^50^	Cross‐sectional	Injuries with no loss of work: IRR 0.867, SE 0.063, *P* < .01 Injuries with work loss: IRR 0.923, SE 0.056, *P* = NS	EAP was associated with a reduction in injuries that resulted in no loss of work, but not injuries that resulted in work loss	Fair
Healthcare Cost	Castro 2000^27^	Historically controlled trial	Workers compensation claims in dollars: Pre‐EAP: 6041.17 (SD: 8705.50) Post‐EAP: 2523.59 (SD: 17 339.19), mean diff: −3517.59 (SD: 3525.04) *P* = .326	Introduction of EAP did not lead to a reduction in total worker compensation claims	Poor
	Sweeney 1995^49^	Controlled interrupted time series	Mental health/chemical dependency claim/costs: EAP user‐non‐user claims: n = 45 pairs, mean difference = −0.05, *P* = .7217 EAP user‐non‐user, cost (dollars), mean difference: n = 45 pairs, *x* = −26.55, *P* = .515	EAP did not result in a significant change in mental health/chemical dependency claims or costs	Fair
Absenteeism	Castro 2000^27^	Historically controlled trial	Sick leaves hours: pre‐EAP: 177.84, Post‐EAP: 64.62, diff: 113.22, SD: 417.757, *P* = .164	Introduction of EAP did not lead to a significant reduction in absenteeism due to sick leaves	Poor
**D. Written workplace drug‐free policy**					
Illicit drug use	Carpenter 2007^26^	Cross‐sectional	Self‐reported marijuana use in the last 30 d: aOR 0.697, SE 0.050, *P* < .01)	Written policy associated with 31% lower self‐reported marijuana use	Poor
	Lee 2011^33^	Cross‐sectional	Misuse of prescription pain relievers. Any drug testing: *β* = 0.2 (0.22) *P* = NS	No association between workplace policy and misuse of prescription pain relievers	Poor
	Miller 2015^39^	Cross‐sectional	Self‐reported non‐medical prescription drug use: (AOR 0.85, 95% CI 0.73‐1.00, *P* = .045)	Written policy associated with 15% lower non‐medical prescription drug use	Fair
	Pidd 2016^44^	Cross‐sectional	Use of illicit drugs in the last 12 mo. AOR, 1.0, 95% CI 0.81‐1.24, *P* = .98	No association between workplace policy and use of illicit drugs	Poor
Work‐related injuries	Waehrer 2016^50^	Cross‐sectional	No work lost injuries: IRR 1.066, SE 0.075, p = NS Injuries with work loss: IRR 1.043, SE 0.043, *P* = NS	A written drug‐free workplace policy was not associated with a reduction in workplace injuries	Fair
**E. Restructuring employee health benefits**					
Healthcare cost	Sturm 2000^48^	Retrospective cohort study	Cost of substance abuse care: Fully managed Behavioral Health organization versus cost‐sharing with workplace: Cost of out‐patient care: regression coefficient = 0.428, *P* < .01 Cost of in‐patient care: regression coefficient = −0.101, *P* = NS	The total cost of out‐patient, but not in‐patients care was lower in organizations that fully contracted out management of substance abuse treatment to Managed Behavioral Health Organizations	Fair
Healthcare utilization	Lo Sasso 2004^34^	Retrospective cohort study	Out‐patient visit utilization: Regression coefficient: −0.069, SE 0.031 *P* < .05 Inpatient treatment days: Regression coefficient: −0.016, SE 0.012, *P* < .0	Increase in co‐payment level was associated with a statistically significant decrease in the number of outpatient and in‐patient treatment visits	Fair
	Sturm 2000^48^	Retrospective cohort study	Access to substance abuse care: Fully managed Behavioral Health organization vs cost sharing with workplace: Access to care: OR = 1.13, *P* = NS	No difference in access to care for employees in organizations that fully contracted out management of substance abuse treatment to Managed Behavioral Health Organizations compared to those who did not	Fair
**F. Combined interventions**					
Illicit drug use	Gómez‐Recasens 2018^32^	Non‐randomized single‐arm study (EE + Drug testing)	Illicit drug use, saliva drug test (Drager drug test) Baseline: 75/1103 (6.8%) Year 1:65/990 (6.6%); baseline vs Year 1, *P* = .332 Year 2:47/700(6.7%); baseline vs Year 2, *P* = .143 Year 3:43/625 (6.9%) baseline vs Year 3, *P* = .108 Year 1 vs Year 2: *P* = .039 Year 2 vs Year 3:*P* = .754,	There was a significant decline in illicit drug use in year 2 compared to year 1, but not at any other time interval	Fair
	Pidd 2016^44^	Cross‐sectional (Written workplace drug‐free policy ± drug testing)	Self‐reported use of illicit drugs in the last 12 mo: aOR, 0.99, 95% CI 0.72‐1.36, *P* = .95	No association between workplace policy ± drug testing and use of illicit drugs	Poor
	Pidd 2016^44^	Cross‐sectional (written workplace drug‐free policy + EE or EAP)	Self‐reported use of illicit drugs in the last 12 mo: aOR, 0.90, 95% CI 0.69‐1.18, *P* = .46	No association between Written workplace policy + EE or (EAP and the use of illicit drugs	Poor
	Pidd 2016^44^	Cross‐sectional (EE + drug testing + Written workplace drug‐free policy ± EAP)	Self‐reported use of illicit drugs in the last 12 mo. aOR, 0.72, 95% CI 0.53‐0.98, *P* = .04	A comprehensive policy was associated with 28% lowers odds of illicit drug use	Poor
Work‐related injuries	Spicer 2005^47^	Controlled Interrupted time‐series analysis (EE + EAP)	Workplace injuries rates: aRR, 0.9984; 95% CI, 0.9975‐0.9994	The combined intervention led to modest (1%) but significant reduction in workplace injuries	Poor
	Miller 2007^40^	Controlled interrupted time series (EE + EAP + Drug testing)	Injuries: Injuries avoided: 824‐849, *P* = .035‐.040	The combined intervention led to significant reduction in workplace injuries	Fair
	Wickizer 2004^51^	Retrospective cohort study (EE + Drug testing + EAP + Supervisor training + Written workplace drug‐free policy)	Injury rates per 100 person‐years (Intervention‐comparison companies): Pre‐intervention = 12.13, 95% CI 11.59‐12.67) During Intervention = 8.80, 95% CI 8.36‐9.23, *P* < .05), Post‐Intervention = 7.36 95% CI 6.44‐8.29, *P* < .05	Organizations that adopted the combined policy experienced a greater decline in workplace injuries (3.3/100 person years)	Fair
	Lockwood 1998^36^	Interrupted time‐series analysis	Workplace accidents: Slope Pre‐intervention = −0.01 Post‐intervention = −0.01 Change in slope: *t*(99) = 0.03, *P* = .976	The combined program did not lead to significant reduction in workplace accidents	Fair
Healthcare Cost	Lockwood 1998^36^	Interrupted time‐series analysis (EE + Drug testing + EAP + Supervisor training + Written workplace drug‐free policy)	Health insurance claims: Slope Pre‐intervention = 3.04 Post‐intervention = 1.57 Change in slope: *t*(50) = −0.55, *P* = .59	The introduction of the combined intervention did not lead to a reduction in health insurance claims	Fair
	Miller 2007^40^ (EE + EAP + drug testing)	Controlled interrupted time series	Injury costs avoided in 1999 (millions of $): 32.7‐33.3, *P* < .01	The combined intervention led to a reduction in the cost of workplace injuries	Fair
Absenteeism	Lockwood 1998^36^	Interrupted time‐series analysis ( EE + Drug testing + EAP + Supervisor training + Written workplace drug‐free policy)	Absenteeism: Slope Pre‐intervention = 1.05 Post‐intervention = −0.94 Change in slope: *t*(61) = −1.79, *P* = .08	The combined program did not lead to a significant reduction in absenteeism	Fair
Productivity	Lockwood 1998^36^	interrupted time‐series analysis (EE + Drug testing + EAP + Supervisor training + Written workplace drug‐free policy)	Productivity: Slope Pre‐intervention = 3.67 Post‐intervention = −3.04 Change in slope: *t*(102) = −1.06, *P* = .29	The combined program did not lead to a significant change in productivity	Fair

Abbreviations: ANOVA, analysis of variance; aOR, adjusted odds ratio; aRR, adjusted relative risk; CI, confidence Interval; *df*, degrees of freedom; EAP, employee assistance program; EE, Employee education; IRR, incidence rate ratio; NS, not statistically significant; OSHA, Occupational Safety and Health Administration of the United; RR, relative risk; SD, standard deviation; SE, standard error.

#### 3.4.1 Employee education

All six evaluations of employee education investigated its effectiveness in reducing employee drug use. Two studies reported a significant reduction in illicit drugs among employees exposed to an educational intervention,^26^, ^28^ while four studies did not find this intervention to be effective.^25^, ^28^, ^29^, ^43^ Three ^25^, ^28^, ^29^, ^43^ of four analyses of RCTs did not find a stand‐alone educational intervention to be effective. Although the fourth RCT^28^ suggested that employee education may lead to a reduction in illicit drug use, the analysis for this outcome lacked methodological rigor. The two remaining studies were analyses of the National Household Surveys on Drug Abuse (NHSDA).^26^, ^39^ One of these studies reported that respondents who endorsed the presence of workplace drug prevention messages were less likely to self‐report marijuana use in 30 days preceding the survey,^26^ while the other did not find an association between workplace education on drug use and self‐reported non‐prescription drug use.^39^ Both studies that suggested that employee education alone was sufficient to reduce drug use^26^, ^28^ had low‐quality assessment scores.

#### 3.4.2 Drug testing

In all, 15 studies evaluated the effectiveness of random, reasonable suspicion, or post‐accident drug testing in the workplace. The most frequent outcome was work‐place injuries.^30^, ^35^, ^37^, ^38^, ^42^, ^45^, ^50^ Five studies investigated the relationship between drug testing and illicit drug use or misuse of prescription drugs,^26^, ^31^, ^33^, ^38^, ^39^ while two investigated the association between drug testing with healthcare cost.^41^, ^42^ One study examined the association between drug testing and productivity.^46^

Two of five studies reported that drug testing was associated with a reduction in drug misuse. Both were cross‐sectional studies, with poor^26^ or fair^31^ quality assessment. Study outcomes were self‐reported marijuana use^26^ or any illicit drug use.^31^ The three other studies did not find any relationship between drug testing and illicit drug use. Two of these were cross‐sectional studies^33^, ^39^ in which no association was found between drug testing and misuse of prescription pain relievers^33^ or non‐medical prescription drug use.^39^ A third study, which analyzed data of a retrospective cohort^38^ did not detect a significant decline in positive urine tests for cocaine and marijuana in a company that switched from non‐random to random drug testing.

Seven studies investigated the association between drug testing and workplace accidents, and two of these studies^35^, ^37^ reported that drug testing was associated with a decline in workplace injuries. In the first of these two studies, the introduction of random drug testing in a company with pre‐employment drug testing led to a significant decline in workplace injuries,^35^ while in the second study, workers randomly selected for drug testing had lower post‐test accident rates when compared to employees who had not had drug testing.^37^ Three studies reported mixed results, indicating that only specific drug‐testing modalities were effective,^30^ or that drug testing was effective for reducing some but not all types of work‐related accidents.^38^, ^50^ In one of these studies, post‐accident drug testing resulted in a decline in Occupational Safety and Health Administration (OSHA) reportable accidents, but reasonable cause drug testing did not have the same effect.^30^ In another study, a switch from non‐random to random drug testing led to a decline in passenger injuries, but not overall accidents among employees in the transport industry.^38^ Lastly, in the study by Waehrer et al,^50^ an association was found between drug testing and injuries resulting in no loss of work, but not injuries associated with loss of work.

In two studies, employee drug testing did not result in a significant reduction in workplace accidents. In one of these studies, there was no significant decline in workplace accidents following the introduction of random drug testing,^42^ while in the other study a combination of pre‐employment and post‐accident and a combination of pre‐employment, post‐accident, random, and suspicion‐based drug testing did not lead to a significant decline in workplace injuries when compared to no drug‐testing program.^45^ Both studies had fair quality assessment ratings.

Two studies investigated the effect of drug testing on healthcare costs. While Morantz and Mas^41^ showed that the adoption of drug testing resulted in a 12% decline in total health claims, Ozminkowski et al^42^ did not find a decline in substance abuse‐related expenditure. Both studies had similar study designs and quality assessment scores. In the only study that investigated the relationship between drug testing and productivity,^46^ any drug testing or specifically random drug testing was associated with a reduction in productivity. The quality of this study was poor, so its findings should be interpreted with caution.

#### 3.4.3 Employee assistance programs

Five studies provided seven evaluations of the effect of EAPs on illicit drug use, work‐related injuries, healthcare costs, or absenteeism. The study by Castro and Lawson,^27^ reported three outcomes: work‐related accidents, healthcare cost, and absenteeism, but had a low‐quality assessment score. Two studies investigated the effect of EAPs on the use of illicit drugs, and one^39^ reported an association between having an EAP and reduced marijuana use, while the other,^26^ with a poor quality score, did not find an independent association between having an EAP program and drug misuse. Both studies were cross‐sectional studies of national surveys, with self‐reported outcomes of marijuana use^26^ or non‐medicinal prescription drug use.^39^

Two studies evaluated the effect of EAPs on workplace accidents. While the study by Castro and Lawson^27^ showed that the introduction of an EAP program led to a significant decline in workplace injuries, the study by Waehrer et al^50^ reported mixed results, and showed an association between EAPs and injuries that resulted in “no loss of work,” but not injuries with “work loss.” The study designs were different: Castro and Lawson^27^ conducted a historically controlled trial, while Waehrer et al^50^ carried out a cross‐sectional study.

None of the two studies that investigated the effectiveness of EAPs in reducing healthcare costs found it to be effective. Sweeney and colleagues^49^ used a matched design to compare manufacturing companies with and without EAPs and did not find a significant difference in the number of claims or the dollar amount of claims between companies with EAPs and those without. Lastly, another analysis in the study by Castro and Lawson^27^ did not show an association between an EAP and total worker compensation claims. There was only one analysis of the effect of an EAP program on absenteeism due to sick leave, and this was reported in the study by Castro and Lawson.^27^ In the cross‐sectional analysis, no association was found between EAPs and absenteeism due to sick leave.

#### 3.4.4 Written drug‐free workplace drug policy

Four^26^, ^33^, ^39^, ^44^ of five studies, all cross‐sectional, investigated the association between a written workplace drug‐free policy and misuse of drugs. Two of these studies reported lower drug misuse (marijuana^26^ or prescription medications^39^), while the other two found no association between written workplace drug‐free policies and misuse of prescription pain relievers^33^ or any illicit drugs.^44^ Three of the four studies were of poor quality,^26^, ^33^, ^44^ while the fourth had fair quality.

One study, also cross‐sectional in design, investigated if there was an association between a written workplace drug‐free policy and work‐related injuries,^50^ and found no association between written drug‐free policy and injuries resulting in loss of work or no‐work‐loss injuries.

#### 3.4.5 Restructuring employee health benefits

Three independent analyses from two retrospective cohort studies, all of fair quality, evaluated the impact of restructuring health benefits on healthcare cost^48^ or utilization.^34^, ^48^ Analyzing health insurance data, Sturm^48^ compared different health insurance plans provided by the same managed health organization but differed in terms of coverage‐fully ensuring contracts versus not. Plans that provided full coverage risk did not have significantly different access rates for any care or any inpatient care. In terms of cost, plans that provided full health coverage were associated with lower out‐patient, but not in‐patient cost.

The second study by Lo Sasso and Lyons^34^ evaluated the impact variation of co‐pay on health services related to employee drug use. The study reported that higher co‐payments were associated with reduced utilization of out‐patient and in‐patient services for patients with drug use problems,^34^ thus having a negative effect on access to care.

#### 3.4.6 Combined interventions

In all, 12 analyses evaluated the effectiveness of a combination of two or more recommended interventions on various work‐related outcomes. Four analyses from two studies had outcomes of drug misuse.^32^, ^44^ One showed that it may be effective,^44^ one had mixed results,^32^ while the remaining two indicated that it was not effective.^44^ Pidd et al,^44^ in a cross‐sectional survey, evaluated various combinations of interventions and reported that the combination of employee education, drug testing, written workplace drug‐free policy, with or without EAP, was associated with a 28% lower odds of self‐reported illicit drug use. In the same study, no association was found between the combination of written workplace drug‐free policy and employee education or EAP, or the combination of written workplace drug‐free policy with or without drug testing, and illicit drug use. The quality of this study was, however, poor.

In a single‐arm study, Gómez‐Recasens et al^32^ examined changes in the yearly proportion of positive saliva drugs screen over 3 years following the introduction of employee education and drug testing. There was a significant decline in year two compared to year one, but not at any other time intervals.

Three^40^, ^47^, ^51^ of four studies reported that a combination of interventions reduced workplace injuries or accidents. The results of a controlled interrupted time‐series analysis^47^ showed a modest but significant decline in workplace injuries after employee education and EAP were introduced to a transportation company. The quality of the study was however poor. In the two other studies, reduction in workplace injuries was reported by Miller et al^40^ and Wickizer et al^51^ in response to the combination of employee education, drug testing, and EAP, or the combination of employee education, drug testing, EAP, supervisor training, and written workplace drug‐free policy, respectively. However, the study by Lockwood et al^36^ did not detect a reduction in workplace accidents after the introduction of a comprehensive policy of employee education, drug testing, EAP, supervisor training, and written workplace drug‐free policy.

Other reported outcomes of combined interventions were healthcare costs,^36^, ^40^ absenteeism,^36^ and productivity.^36^ Of these, only the study by Miller et al^40^ reported a positive outcome, with the combination of employee education, drug testing, and EAP, resulting in a significant decline in the cost attributable to workplace injuries.

## 4 DISCUSSION

We have provided an updated, systematic assessment of the effectiveness of currently recommended interventions for employers to prevent or reduce the adverse effects of opioids and related drugs. Building on previous reviews,^9^, ^10^, ^11^, ^12^, ^13^, ^14^ we adopted a systematic approach and included all currently recommended interventions to insulate employees from drug use, and included all outcomes we considered will be important to both employers and employees. However, similar to what was observed in previous reviews, most of the studies were methodologically weak, providing a poor evidence base to access the efficacies of these interventions.

In light of the opioid epidemic and increasing legalization of marijuana,^52^ the rising incidence of substance use disorders and its impact on the workforce is a serious concern.^52^, ^53^, ^54^, ^55^ Yet, of the 27 studies identified in this research, only seven were published in the past decade. Of these seven, four were cross‐sectional analyses of national survey data. Of the three remaining studies from the past decade, when the effects of the crisis were first being detected, only one study was based in the United States.^45^ Coincidently, this study has the highest quality assessment score of all 27 publications. Unfortunately, this single piece of recent evidence is not particularly useful guidance for employers. The mixed results of this review may be disappointing to employers looking for clear guidance on interventions to adopt to address substance use. Overall, our findings suggest that the interventions may work in some contexts, but not others, which highlights the need for mixed methods evaluations of employer‐led interventions. Such studies would provide evidence about the contexts in which the interventions are more likely to succeed.

Despite these shortcomings, the results from the identified studies indicate that work‐related injuries or accidents may be more sensitive to the effects of the evaluated workplace interventions. Three^40^, ^47^, ^51^ of four combined interventions with outcomes of work‐related injuries reported a significant decline in injuries. Five^30^, ^35^, ^37^, ^38^, ^50^ of seven studies reported that drug testing might reduce workplace injuries, and both studies that evaluated the impact of EAP^27^, ^50^ reported lower accidents associated with EAP. Outcome data related to workplace injuries may also be more reliable than data on drug use as the former may be pulled from standard documentation required by OSHA, and the latter from self‐reports.

In response to the opioid epidemic, our goal was to provide a comprehensive review of the effectiveness of interventions that employers can deploy to mitigate the adverse workplace effects of opioids. Despite our efforts to achieve this goal, the limitations of our review need to be considered. Because of the variations in study designs, effect measures, and outcomes, we were unable to conduct a meta‐analysis. However, given the poor quality of identified studies, this may not have a significant effect on the overall conclusions. Also, our choice for the Downs and Black was based on its rigor in assessing the quality of both RCTs and non‐RCTs and its wide use.^20^, ^21^, ^22^, ^23^ Using a different tool may have produced different results related to study quality. Despite these limitations, to the best of our knowledge, this is the most comprehensive synthesis of the effectiveness of currently recommended interventions that can be instituted by employers for addressing substance misuse in the workforce.

We suspect that many employers have implemented the interventions described here,^6^ but few employers may have evaluated and published the results. It is not surprising, given that these research activities are not central to the core business of most employers and that many employers might not be familiar with conducting and publishing rigorous research. There is an opportunity for employer‐researcher partnerships to help with evaluations of these employer‐led interventions. Researchers may help employers identify interventions, evaluate interventions, and bridge the gap between what is known and what is practiced. There is also the potential for greater partnerships between public health agencies and large employers in efforts to prevent and reduce substance use disorders. Large employers have a financial incentive to reduce substance abuse in their workers. They also have the opportunity to reach large numbers of people both by intervening directly with their employees and indirectly through the families and dependents of their employees. Future partnerships between large employers and researchers could strengthen the knowledge base about effective interventions and guide other employers to help their workforce.

## 5 CONCLUSIONS

In conclusion, our systematic review found no rigorous evaluations of employer‐led efforts to prevent or reduce the ill effects of substance abuse disorder. As a result, there are limited evidence‐based strategies for employers to consider for addressing substance use. More employer‐led experimentation, employer‐researcher and employer‐public health partnerships, and mixed methods evaluations may help to expand the evidence base. Based on the available evidence, recommended interventions may reduce workplace injuries, but require more rigorous confirmatory research.

## DISCLOSURE

*Approval of the research protocol*: N/A. *Informed consent*: N/A. *Registry and the registration no. of the study/trial*: The review protocol is registered in the International prospective register of systematic reviews, PROSPERO (Registration number: CRD42019132681); *Animal studies*: N/A; *Conflict of interest*: All authors declare no competing interest.

## AUTHOR CONTRIBUTIONS

MCM was responsible for conceptualization. All authors were involved in the study design. MOA, LCM, CBI, and ASR were responsible for data extraction, while all authors were involved in data analysis. MOA, LCM, and CBI were responsible for writing the initial draft of the manuscript, and all authors were involved in reviewing and editing.

## Supporting information

Supplementary Material

Supplementary Material
