# Supplementary material for: A systematic review of the effectiveness of employer‐led interventions for drug misuse
Source: J Occup Health. 2020 Jun 13;62(1):e12133. doi: 10.1002/1348-9585.12133 (PMC7293184; doi:10.1002/1348-9585.12133)
Supplement: Supplementary file 1 — Supplementary Material [file JOH2-62-e12133-s001.docx]

**Supplementary Materials: APPENDIX**

**Title: A systematic review of employer-led interventions to prevent substance use disorders**

**Authors:**

Maxwell O. Akanbi. MD, MSCI^1^

Cassandra B. Iroz M.S^1^

Linda C. O'Dwyer MA, MSLIS^2^

Adovich S. Rivera. MD^1^

Megan Colleen McHugh. PhD ^1, 3^

**Affiliations:**

^1^Institute for Public Health and Medicine, Feinberg School of Medicine, Northwestern University, Chicago, USA

^2^ Galter Health Sciences Library and Learning Center, Northwestern University, Feinberg School of Medicine, 303 East Chicago Avenue Chicago, Illinois 60611

^3^Department of Emergency Medicine, Feinberg School of Medicine, Northwestern University

**APPENDIX A: Search Strategies**

**PubMed search, June 2016**

(misuse OR abuse OR overuse OR “prescription drug misuse”[mesh] OR "Inappropriate Prescribing"[Mesh])

AND ("Analgesics, Opioid"[Mesh] OR "Analgesics, Opioid" [Pharmacological Action] OR opioid* OR opiate* OR narcotic*)

AND (“Narcotic bowel syndrome”[tiab] OR Abdominal[tiab] OR gastrointestinal[tiab] OR "Inflammatory Bowel Diseases"[Mesh] OR “Inflammatory bowel disease”[tiab] OR "Irritable Bowel Syndrome"[Mesh] OR “Irritable bowel syndrome”[tiab] OR "Pancreatitis"[Mesh] OR Pancreatitis[tiab] OR “functional gastrointestinal disorder”[tiab] OR "Pelvic Pain"[Mesh] OR “pelvic pain”[tiab] OR “non-cardiac chest pain”[tiab] OR "Gastrointestinal Diseases"[Mesh] OR “centrally mediated abdominal pain syndrome”[tiab] OR ("Dyspepsia"[Mesh] AND functional[tiab]) OR “functional dyspepsia”[tiab] OR ("Heartburn"[Mesh] AND functional[tiab]) OR “functional heartburn”[tiab] OR “functional biliary sphincter”[tiab] OR “functional gallbladder disorder”[tiab] OR "Biliary Dyskinesia"[Mesh] OR “biliary dyskinesia”[tiab] OR "Sphincter of Oddi Dysfunction"[Mesh] OR “sphincter of oddi”[tiab] OR "Constipation"[Mesh] OR “constipation”[tiab] OR “abdominal wall pain”[tiab] OR "Abdominal Pain"[Mesh] OR “chronic abdominal pain”[tiab] OR "Crohn Disease"[Mesh] OR “crohn's disease”[tiab] OR “crohn disease”[tiab] OR “crohns disease”[tiab] OR "Colitis, Ulcerative"[Mesh] OR “ulcerative colitis”[tiab] OR “colitis gravis”[tiab] OR “idiopathic protocolitis”[tiab] OR "Gastroparesis"[Mesh] OR “gastroparesis”[tiab] OR “gastropareses”[tiab] OR “gastric stasis”[tiab] OR “gastric stases”[tiab])

**n = 693**

**Embase search, June 2016**

'drug misuse'/exp OR 'multiple drug abuse'/exp OR 'drug abuse pattern'/exp OR 'potentially inappropriate medication'/exp OR misuse:ti,ab OR abuse:ti,ab OR overuse:ti,ab

AND
'opiate'/exp OR opioid*:ab,ti OR opiate*:ab,ti OR narcotic*:ab,ti

AND

'gastrointestinal disease'/exp OR 'inflammatory bowel disease'/exp OR 'irritable colon'/exp OR 'gastrointestinal motility disorder'/exp OR 'gastrointestinal reflux'/exp OR 'intestine function disorder'/exp OR 'stomach function disorder'/exp OR 'pancreatitis'/exp OR 'pelvic pain'/exp OR 'noncardiac chest pain'/exp OR 'narcotic bowel syndrome':ab,ti OR abdominal:ab,ti OR gastrointestinal:ab,ti OR 'inflammatory bowel disease':ab,ti OR 'irritable bowel syndrome':ab,ti OR pancreatitis:ab,ti OR 'functional gastrointestinal disorder':ab,ti OR 'pelvic pain':ab,ti OR 'non-cardiac chest pain':ab,ti OR ‘centrally mediated abdominal pain syndrome’:ti,ab OR ('dyspepsia'/exp AND functional:ti,ab) OR ‘functional dyspepsia’:ti,ab OR ('heartburn'/exp AND functional:ti,ab) OR ‘functional heartburn’:ti,ab OR ‘functional biliary sphincter’:ti,ab OR ‘functional gallbladder disorder’:ti,ab OR 'bile duct dyskinesia'/exp OR ‘bile duct dyskinesia’:ti,ab OR ‘biliary dyskinesia’:ti,ab OR 'Oddi sphincter'/exp OR ‘sphincter of oddi’:ti,ab OR 'constipation'/exp OR ‘constipation’:ti,ab OR ‘abdominal wall pain’:ti,ab OR 'abdominal pain'/exp OR ‘chronic abdominal pain’:ti,ab OR 'Crohn disease'/exp OR ‘crohn disease’:ti,ab OR ‘crohns disease’:ti,ab OR 'ulcerative colitis'/exp OR ‘ulcerative colitis’:ti,ab OR ‘colitis gravis’:ti,ab OR ‘idiopathic protocolitis’:ti,ab OR 'stomach paresis'/exp OR ‘gastroparesis’:ti,ab OR ‘gastropareses’:ti,ab OR ‘gastric stasis’:ti,ab OR ‘gastric stases’:ti,ab

**N = 1049**

**CENTRAL search, June 2016**

Date Run: 24/06/16 18:01:33.451

Description:

ID Search Hits

#1 MeSH descriptor: [Prescription Drug Misuse] explode all trees 111

#2 MeSH descriptor: [Inappropriate Prescribing] explode all trees 71

#3 misuse:ti,ab or abuse:ti,ab or overuse:ti,ab 5268

#4 #1 or #2 or #3 5426

#5 MeSH descriptor: [Analgesics, Opioid] explode all trees 5738

#6 opioid*:ti,ab or opiate*:ti,ab or narcotic*:ti,ab 11669

#7 MeSH descriptor: [Gastrointestinal Diseases] explode all trees 26661

#8 "gastrointestinal disease":ti,ab or "inflammatory bowel disease":ti,ab or "irritable colon":ti,ab or "gastrointestinal motility disorder":ti,ab or "gastrointestinal reflux":ti,ab or "intestine function disorder":ti,ab or "stomach function disorder":ti,ab or "pancreatitis":ti,ab or "pelvic pain":ti,ab or "noncardiac chest pain":ti,ab or "narcotic bowel syndrome":ab,ti or abdominal:ab,ti or gastrointestinal:ab,ti or "inflammatory bowel disease":ab,ti or "irritable bowel syndrome":ab,ti or pancreatitis:ab,ti or "functional gastrointestinal disorder":ab,ti or "pelvic pain":ab,ti or "non-cardiac chest pain":ab,ti or "centrally mediated abdominal pain syndrome":ti,ab or "functional dyspepsia":ti,ab or "functional heartburn":ti,ab or "functional biliary sphincter":ti,ab or "functional gallbladder disorder":ti,ab or "bile duct dyskinesia" or "bile duct dyskinesia":ti,ab or "biliary dyskinesia":ti,ab or "Oddi sphincter" or "sphincter of oddi":ti,ab or "constipation" or "constipation":ti,ab or "abdominal wall pain":ti,ab or "abdominal pain" or "chronic abdominal pain":ti,ab or "Crohn disease" or "crohn disease":ti,ab or "crohns disease":ti,ab or "ulcerative colitis" or "ulcerative colitis":ti,ab or "colitis gravis":ti,ab or "idiopathic protocolitis":ti,ab or "stomach paresis" or "gastroparesis":ti,ab or "gastropareses":ti,ab or "gastric stasis":ti,ab or "gastric stases":ti,ab 44203

#9 #5 or #6 14798

#10 #7 or #8 63495

#11 #4 and #9 and #10 28

= 9 Cochrane SRs

=19 trials

**n = 28**

**Web of Science search, June 2016**

(misuse OR abuse OR overuse OR “prescription drug misuse” OR "Inappropriate Prescribing")

AND (opioid* OR opiate* OR narcotic*)

AND (“Narcotic bowel syndrome” OR Abdominal OR gastrointestinal OR "Inflammatory Bowel Diseases" OR “Inflammatory bowel disease” OR "Irritable Bowel Syndrome" OR “Irritable bowel syndrome” OR "Pancreatitis" OR “functional gastrointestinal disorder” OR “pelvic pain” OR “non-cardiac chest pain” OR "Gastrointestinal Diseases" OR “centrally mediated abdominal pain syndrome” OR “functional dyspepsia” OR “functional heartburn” OR “functional biliary sphincter” OR “functional gallbladder disorder” OR "Biliary Dyskinesia" OR “sphincter of oddi” OR "Constipation" OR "Crohn Disease" OR “crohn’s disease” OR “crohns disease” OR “ulcerative colitis” OR “colitis gravis” OR “idiopathic protocolitis” OR "Gastroparesis" OR “gastroparesis” OR “gastropareses” OR “gastric stasis” OR “gastric stases” OR “irritable colon” OR “gastrointestinal motility disorder” OR “gastrointestinal reflux” OR “intestine function disorder” OR “stomach function disorder”)

n= 225 citations

**Scopus search, June 2016**

( ( TITLE-ABS-KEY ( ( misuse  OR  abuse  OR  overuse  OR  "prescription drug misuse"  OR  "Inappropriate Prescribing" ) )  AND  TITLE-ABS-KEY ( ( opioid*  OR  opiate*  OR  narcotic* ) ) ) )  AND  ( TITLE-ABS-KEY ( "Narcotic bowel syndrome"  OR  abdominal  OR  gastrointestinal  OR  "Inflammatory Bowel Diseases"  OR  "Inflammatory bowel disease"  OR  "Irritable Bowel Syndrome"  OR  "Irritable bowel syndrome"  OR  "Pancreatitis"  OR  "functional gastrointestinal disorder"  OR  "pelvic pain"  OR  "non-cardiac chest pain"  OR  "Gastrointestinal Diseases"  OR  "centrally mediated abdominal pain syndrome"  OR  "functional dyspepsia"  OR  "functional heartburn"  OR  "functional biliary sphincter"  OR  "functional gallbladder disorder"  OR  "Biliary Dyskinesia"  OR  "sphincter of oddi"  OR  "Constipation"  OR  "Crohn Disease"  OR  "crohn's disease"  OR  "crohns disease"  OR  "ulcerative colitis"  OR  "colitis gravis"  OR  "idiopathic protocolitis"  OR  "Gastroparesis"  OR  "gastroparesis"  OR  "gastropareses"  OR  "gastric stasis"  OR  "gastric stases"  OR  "irritable colon"  OR  "gastrointestinal motility disorder"  OR  "gastrointestinal reflux"  OR  "intestine function disorder"  OR  "stomach function disorder" ) )

**n=1241**

**APPENDIX B: Major Drugs of Abuse in the US**

**(Source: National Survey on Drug Use and Health 2016)**

1. Marijuana
2. Prescription pain relievers (See Figure on the next page) (Includes prescription Opioids)
   1. Hydrocodone Products
   2. Oxycodone Products
   3. Tramadol Products
   4. Codeine Products
   5. Morphine Products
   6. Fentanyl Products
   7. Buprenorphine Products
   8. Oxymorphone Products
   9. Demerol®
   10. Hydromorphone Products
   11. Methadone
3. Prescription tranquilizers/ sedatives
   1. Pentobarbital
   2. Xanax (Alprazolam)
   3. Limbitrol (Chlordiazepoxide)
   4. Valium (Diazepam)
   5. Ativan (Lorazepam)
   6. Halcion (Triazolam)
   7. Lunesta (Eszopiclone)
   8. Sonata (Zaleplon)
   9. Ambien (Zolpidem)
4. Cocaine
5. Prescription Stimulations
   1. Dextroamphetamine (Dexedrine®)
   2. Dextroamphetamine/amphetamine combination product (Adderall®)
   3. Methylphenidate (Ritalin®, Concerta®).
6. Hallucinogens
   1. lysergic acid diethylamide (LSD_
   2. Phencyclidine (PCP)
   3. Peyote
   4. Mescaline
   5. psilocybin mushrooms,
   6. “Ecstasy” (MDMA or “Molly”)
   7. Ketamine
   8. DMT/AMT/“Foxy,
   9. *Salvia divinorum*
7. Methamphetamine
8. Heroine
